# 

**Published:** 1957-10

**Authors:** 


MEDICAL JOURNAL OF THE SOUTH-WEST

*(Incorporating the Bristol Medico-Chirurgical Journal)*

Established 1883

VOL. 72 (iv) October, 1957 NO. 266

### Contents

| | | Page |
|---|---|---|
| CLINICAL PATHOLOGY AND GENERAL PRACTICE: | *Dr. H. J. Gibson* | 109 |
| TOWARDS BETTER HEALTH AT WORK | *Dr. T. G. Faulkner Hudson* | 117 |
| CHILD ART . . . . . | *Mr. D. E. Milner* | 125 |
| ACUTE NECROSIS OF THE LIVER IN PREGNANCY | *Clinical Pathological Conference* | 129 |
| MEMBERS OF THE BRISTOL MEDICO-CHIRURGICAL SOCIETY . | | 133 |
| SUBSCRIBERS TO THE "JOURNAL" . . . . . | | 140 |
| NEWS FROM SOCIETIES . . . . . . | | 141 |
| APPOINTMENTS . . . . . . . . | | 145 |

*PUBLISHED QUARTERLY*

*ANNUAL SUBSCRIPTION* 16/- *POST FREE*

PUBLISHED UNDER THE AUSPICES OF THE
BRISTOL MEDICO-CHIRURGICAL SOCIETY

### Editorial Committee

Dr. J. E. Cates, Department of Medicine, Bristol Royal Infirmary. *Editor.*
Dr. N. J. Brown, Southmead Hospital, Bristol. *Assistant Editor.*
Dr. J. Macrae, Ham Green Hospital.
Dr. R. C. Wofinden, Central Clinic, Bristol.
Mr. A. E. S. Roberts, Medical Library, University of Bristol. *Secretary.*

*Local Correspondents*

*Bath* . . Mr. A. D. Bateman, 3 The Circus, Bath.
*Exeter* . . Dr. L. N. Jackson, Mount Jocelyn, Crediton, Devon.
*Gloucester* . Dr. R. F. Jarrett, 9 College Green, Gloucester.
*Plymouth* . Dr. C. R. Croft, Lexden, Hartley Av., Plymouth.
*Taunton* . Dr. M. McAlley, 1 The Crescent, Taunton.
*Torquay* . Dr. A. Robinson Thomas, 20 Devon Square, Newton Abbot, Devon.
*Truro* . . Dr. F. D. M. Hocking, St. Andrews, Perranporth, Cornwall.
*Yeovil* . . Mr. J. A. Vere Nicoll, Knapp House, Yeovil, Somerset.

### NOTICE TO CONTRIBUTORS

Original articles are invited, provided they have not been published elsewhere. Notes of forthcoming events, meetings held, degrees conferred, staff appointments, and other local news are welcomed. The editorial committee reserves the right to make literary corrections.

Illustrations should always be supplied ready for reproduction. All re-touchings or other operations required will be charged to the author. Line drawings should be drawn in Indian ink. We regret that we must ask authors to pay for making blocks, if this is necessary.

J. W. ARROWSMITH LTD., WINTERSTOKE ROAD,
BRISTOL.

### MEMBERS OF THE BRISTOL MEDICO-CHIRURGICAL SOCIETY*

Mr. A. W. ADAMS, Rodney House, Clifton, Bristol 8.

Dr. G. R. AIRTH, Trevi House, Silverhill Brake, Rudgeway, Bristol.

Mr. A. C. AKEHURST, Royal Infirmary, Bristol.

Dr. J. ALDREN, 39 Oakfield Road, Bristol 8.

Mr. G. L. ALEXANDER, Springhill, Leigh Woods, Bristol.

Dr. A. F. ALFORD, Ministry of Education, Special Services Branch, 36–38 Berkeley Square, London, W.1.

Mr. MICHAEL ALMS, Royal Infirmary, Bristol.

Dr. C. T. ANDREWS, Bryn, Falmouth Road, Truro.

Dr. J. APLEY, Royal United Hospital, Bath.

Dr. K. P. ARNEY, 43 Regent Street, Kingswood, Bristol.

Dr. L. G. BACKHURST, 41 Woodbridge Road, Knowle, Bristol 4.

Dr. W. BAIN, 2 Rylestone Grove, Stoke Bishop, Bristol 9.

Dr. V. BAKER, 72 Locking Road, Weston-super-Mare.

Dr. P. B. BAILEY, 51 Sussex Place, Bristol.

Dr. R. F. BARBOUR, Hill House, Iron Acton, Glos.

Dr. J. S. BARKER, 566 Fishponds Road, Bristol.

Dr. R. H. BARNFIELD, The Field House, Brandon Hill, Bristol 8.

Mr. C. BARTLETT, "Rosebank", Henbury, Bristol.

Mr. J. BASTOW, Circus House, The Circus, Bath.

Dr. A. D. BATEMAN, 11 The Circus, Bath.

Dr. U. BAXTER, Lime Breach, Camp Lane, Clapton-in-Gordano, Somerset.

Dr. V. T. BAXTER, Lime Breach, Camp Lane, Clapton-in-Gordano, Somerset.

Dr. D. L. BAYLEY, 61 Westbury Road, Bristol 9.

Dr. R. W. BAZELEY, The Hewletts, Radstock, Bath.

Dr. D. H. BEATON, 8 Richmond Park Road, Bristol 8.

Dr. J. BEATSON, 320 Canford Lane, Bristol 9.

Dr. H. BECK, 15 North View, Staple Hill, Bristol.

Dr. A. A. K. BEDDOE, Manor Farm House, Horfield, Bristol 7.

Dr. R. J. I. BELL, 5a Oakfield Road, Bristol 8.

Mr. R. BELSEY, Frenchay Hospital, Bristol.

Dr. J. BENN, 9 Haines Hill, Taunton.

Dr. M. O. BENNETT, 37 West View Road, Keynsham, Somerset.

Dr. ELIZABETH M. BERRY, 58 The Ridgway, Long Ashton, Bristol.

Dr. R. J. A. BERRY, 3c All Saints Road, Bristol 8.

Dr. J. E. BEVISS, 8 Queen's Parade, Bath.

Dr. J. A. BIRRELL, 1 Victoria Square, Bristol 8.

Dr. G. W. R. BISHOP, Bloomfield House, High Street, Shepton Mallet, Somerset.

Dr. MONICA BISHOP, Bedford General Hospital, Kempston Road, Bedford.

Mr. W. R. BLACK, Warberry, Kew Road, Weston-super-Mare.

Mr. B. E. BLAIR, 11 The Circus, Bath.

Mr. D. C. BODENHAM, 31 Old Sneed Road, Stoke Bishop, Bristol 9.

Dr. F. H. BODMAN, 3 Litfield Place, Clifton, Bristol 8.

Dr. M. BODYCOMBE, 288 Bishopsworth Road, Bristol 3.

Dr. D. BOLT, 26 Haverstock Road, Knowle, Bristol 4.

Dr. J. R. BOLTON, West Lodge, 61 Englishcombe Lane, Bath.

Dr. B. J. BOULTON, 48 Kellaway Avenue, Bristol 6.

Mr. H. BOURNS, 16 Ormerod Road, Stoke Bishop, Bristol 9.

Dr. N. J. BOWN, The Corner, Nailsea, Somerset.

Dr. E. G. BRADBEER, 21 Rayleigh Road, Westbury-on-Trym, Bristol.

Mr. J. W. BRADBEER, 10 Usk Road, Llanishen, Cardiff.

Dr. A. G. W. BRANCH, Tor Cleve, 10 Dial Hill Road, Clevedon, Somerset.

Dr. H. W. BRASSINGTON, 17 Fairfield Road, Montpelier, Bristol 6.

Dr. W. P. BRINCKMAN, 2 Clifton Park, Bristol 8.

Dr. R. B. BRITTON, 33a Whiteladies Road, Bristol 8.

Dr. W. L. BROADFOOT, Stapleton Hospital, Fishponds, Bristol.

Professor R. J. BROCKLEHURST, Department of Physiology, The University, Bristol 8.

Mr. C. A. BROWN, 16 Grove Road, Coombe Dingle, Bristol.

Dr. D. D. BROWN, Clyde House, Clyde Road, Frampton Cotterell, near Bristol.

Dr. N. BROWN, 6 The Avenue, Clifton, Bristol 8.

Dr. N. F. W. BRUETON, 31 The Dell, Westbury-on-Trym, Bristol.

* Please report any errors or omissions to Mr. A. E. S. Roberts, Medical Library, The University, Bristol.

Dr. E. Bryant, 17 Julian Road, Bristol 9.
Dr. A. E. Burton, 24 The Circus, Bath.
Dr. M. H. Buston, 6 Cotham Hill, Bristol 6.
Mr. T. J. Butler, Downderry, Mariner's Drive, Stoke Bishop, Bristol 9.
Dr. F. J. Cairns, Devon House, Whitehall Road, Bristol 5.
Dr. D. Cameron, 1 Dean Lane, Southville, Bristol 3.
Dr. M. Cameron, 1 Dean Lane, Southville, Bristol 3.
Dr. W. Cameron, 1 Dean Lane, Southville, Bristol 3.
Dr. A. M. G. Campbell, 79 Pembroke Road, Bristol 8.
Dr. H. Caplan, 3 Cotham Road, Bristol 6.
Mr. W. M. Capper, 131 Pembroke Road, Bristol 8.
Dr. R. S. Carey, 502 Bath Road, Brislington, Bristol 4.
Dr. H. H. Carleton, Robin Hill, West Hill, Wraxall, Somerset.
Dr. T. G. Carmichael, 179 Wick Road, Bristol 4.
Dr. W. H. K. Carpenter, Beaufort House, Stapleton, Bristol.
Dr. J. Y. Carter, 89 Coldharbour Road, Bristol 6.
Dr. A. H. Casson, Hill House, Chipping Sodbury, Glos.
Dr. J. E. Cates, 22 Eastover Close, Westbury-on-Trym, Bristol.
Dr. J. H. Challenger, Avon Prior, Durley Park, Keynsham, Somerset.
Dr. W. A. Chapman, 41 Salisbury Road, Bristol 6.
Mr. H. Chitty, Naish House, Clapton-in-Gordano, Somerset.
Mr. K. Chitty, The Cottage, Naish House, Clapton-in-Gordano, Somerset.
Mr. L. E. Claremont, 28 Victoria Square, Bristol 8.
Dr. D. O. Clark, Mendip Croft, Bleadon, Weston-super-Mare.
Dr. A. D. Clarke, 40 Eastfield, Westbury-on-Trym, Bristol.
Dr. S. K. R. Clarke, Harley Lodge, Clifton Park, Bristol 8.
Dr. F. Clifton, 28 Victoria Square, Bristol 8.
Dr. J. F. Coates, Friars Gate, Bridgwater, Somerset.
Dr. P. C. Collyns, Ash Hayes, Nailsea, Somerset.
Mr. R. V. Cooke, Litfield House, Clifton, Bristol 8.
Dr. N. Cook, 590 Wells Road, Bristol 4.
Dr. D. J. Cooper, 157 Bradford Road, Combe Down, Bath.
Dr. B. Corner, 3 Elton House, Rodney Place, Bristol 8.
Dr. J. A. Cosh, 6 Woodland Place, Bathwick Hill, Bath.
Dr. D. G. Cossham, Kingsleigh, Cirencester.
Dr. H. H. Coulter, 90 Reedley Road, Bristol 9.
Dr. R. M. Courtney, Manor House, Southmead Road, Bristol 9.
Dr. A. A. Craig, 35 Queen's Court, Bristol 8.
Dr. N. S. Craig, Cecil Lodge, The Avenue, Sneyd Park, Bristol 9.
Dr. R. A. Craig, 15 Old Sneyd Park, Bristol 9.
Dr. G. E. Cree, Maudlin Street Clinic, Bristol 2.
Dr. B. A. Crook, The Laurels, Timsbury, Bath.
Mr. H. J. Croot, Department of Surgery, Makerere College, Kampala, Uganda.
Dr. L. H. Crosby, 43 Nevil Road, Bristol 7.
Dr. R. S. Crow, "Greenhayes", Chestnut Road, Long Ashton, Bristol.
Dr. G. I. Culank, 113 Nags Head Hill, St. George, Bristol 5.
Dr. S. Curwen, 9 Clifton Park, Bristol 8.
Mr. G. A. Dalton, Bradley Avenue, Winterbourne, Glos.
Dr. U. Damrel, Cadbury Lodge, Claverham, near Bristol.
Professor A. I. Darling, Dental Hospital, Bristol 1.
Dr. K. Datta, 31 Copley Gardens, Bristol 7.
Dr. D. H. Davies, 23 Pembroke Road, Bristol 8.
Dr. D. T. Davies, 6 The Dell, Westbury-on-Trym, Bristol 9.
Dr. E. D. D. Davies, Battledown Court, Cheltenham, Glos.
Dr. N. Delaney, The Meade, Bishopsworth, Somerset.
Dr. M. Dickson, 47 Canynge Road, Clifton, Bristol 8.
Dr. C. Dix, 14 Mortimer Road, Bristol 8.
Dr. M. Dixon, 7 Windsor Terrace, Bristol 8.
Dr. W. E. Dobson-Smith, 121 High Street, Hanham, Bristol.
Dr. A. A. Dowling, 87 Parry's Lane, Bristol 9.
Dr. J. R. Duerden, 4 Cecil Road, Bristol 8.
Dr. K. P. Duncan, 9a Quiet Street, Bath.
Dr. H. R. Dutton, 405 Stapleton Road, Bristol 5.
Dr. D. F. Early, Fishponds Hospital, Bristol.
Dr. H. J. Eastes, Bank House, Marshfield, Chippenham, Wilts.
Dr. Z. Eastes, Bank House, Marshfield, Chippenham, Wilts.
Dr. J. H. C. Eglinton, Street, Somerset.
Dr. J. Elliott, 29 Old Sneed Avenue, Bristol 9.
Dr. W. H. G. Elliott, 100 Redland Road, Bristol 6.

Dr. W. T. Ellis, Tregenna House, Shirehampton, Bristol.

Dr. R. Esler, 19 Southmead Road, Bristol.

Dr. Jean Eustace, 47 Englishcombe Lane, Bath.

Dr. C. D. Evans, Litfield House, Clifton, Bristol 8.

Dr. G. M. Evans, 117 Ashley Road, Bristol.

Dr. J. Evans, 326 Wells Road, Bristol 4.

Dr. C. C. Ewing, Eye Hospital, Bristol 1.

Mr. A. L. Eyre-Brook, Litfield House, Clifton, Bristol 8.

Dr. E. M. Eyre-Brook, Druids Mead, Shirehampton Road, Stoke Bishop, Bristol 9.

Mr. H. D. Fairman, 9 Clifton Park, Bristol 8.

Dr. J. L. Faull, 61 Cotham Brow, Bristol 6.

Dr. G. R. Fells, 17 Sion Hill, Clifton, Bristol 8.

Dr. R. R. Fells, 17 Mortimer Road, Clifton, Bristol 8.

Dr. G. L. Feneley, Avon Lodge, 8 Parry's Lane, Bristol 9.

Dr. E. J. Field, Department of Anatomy, The University, Bristol 8.

Dr. J. G. Field, Abbotshill, Durley Park, Keynsham, Near Bristol.

Dr. H. F. M. Finzel, The Beeches, St. Anne's Park, Bristol 4.

Mr. G. M. Fitzgibbon, Hill House, Wickwar, Glos.

Dr. A. C. Fleming, Hortham Hospital, Almondsbury, Glos.

Dr G. L. Foss, 3 Litfield Place, Clifton, Bristol 8.

Dr. H. W. S. Francis, 26 Barley Croft, Westbury-on-Trym, Bristol.

Dr. D. H. Fox, 27 Rannoch Road, Filton, Bristol 7.

Dr. A. D. Fraser, Cornerways, Dragon's Well Road, Brentry, Bristol.

Dr. C. J. Fraser, 20 Dragon's Well Road, Brentry, Bristol.

Dr. S. Fraser, 20 Dragon's Well Road, Brentry, Bristol.

Mr. J. Freeman, 22 Downs Park West, Durdham Down, Bristol 6.

Dr. H. L. Fuller, 9 Gay Street, Bath.

Dr. P. K. Ganguli, Southmead Hospital, Bristol 9.

Mr. R. R. Garden, Camp House, Clifton Down, Bristol 8.

Dr. E. T. Garthwaite, 55 Bridgwater Road, Bristol 3.

Dr. K. H. Gaskell, The Old House, Frenchay, Bristol.

Dr. N. Gerrish, 2 Station Road, Keynsham, Near Bristol.

Dr. B. F. Gibson, Nare House, Birchwood Road, Bristol 4.

Dr. M. D. Gibson, 23 Clifton Wood Road, Bristol 8.

Dr. W. A. Gillespie, 2 Church Avenue, Stoke Bishop, Bristol 9.

Mr. S. Glaser, Sunnyside, Weston Road, Bath.

Dr. H. M. Golding, 10 Barton Hill Road, Bristol 5.

Dr. I. R. S. Gordon, Yew Tree Farm, Chew Stoke, Somerset.

Dr. A. P. Gorham, 53 Westbury Road, Westbury-on-Trym, Bristol.

Dr. J. M. Graham, 1 Manor Road, Fishponds, Bristol.

Dr. D. R. Gray, Lostwood, Langford, Near Bristol.

Dr. E. P. Griffiths, B.A.C. (Aero Engines), Filton House, Bristol.

Mr. W. Hedley Hall, High View, Limpley Stoke, Near Bath.

Mr. E. J. Hargadon, Hambrook Hospital, Hambrook, Glos.

Dr. Jane Hargreaves, St. Martin's Hospital, Bath.

Dr. J. H. T. Harrington, 74 Parry's Lane, Bristol 9.

Dr. H. E. Harris, The Mount, Halse, Taunton.

Dr. G. Hartill, Stone House, Chipping Sodbury, Glos.

Dr. G. Hartley, 19 Queen's Court, Clifton, Bristol 8.

Mr. F. Hector, 1 Victoria Square, Bristol 8.

Dr. R. Hemphill, Molitor, Barrow Gurney, Near Bristol.

Dr. A. G. Heron, 237 Gloucester Road, Bristol 7.

Professor T. F. Hewer, Canynge Hall, Bristol 8.

Dr. M. P. Hickey, 3 Mortimer Road, Clifton, Bristol 8.

Dr. L. C. Hill, 18 The Circus, Bath.

Dr. P. G. Hill, 45 Stoke Hill, Stoke Bishop, Bristol 9.

Dr. H. L. Hoffman, 26 The Circus, Bath.

Dr. J. M. Hoffman, Turley Villa, Near Bradford-on-Avon.

Dr. C. M. Hogg, High Street, Oldland Common, Near Bristol.

Dr. L. C. Holland, Pinckney's Farm House, Durrington, Salisbury.

Dr. W. A. L. Holland, 134 Stoke Lane, Westbury-on-Trym, Bristol.

Dr. J. A. G. Holt, 109 Falconwood Road, Addington, Surrey.

Dr. J. A. Hooker, Northlands, Cowes, Isle of Wight.

Mr. R. E. Horton, Royal Infirmary, Bristol 2.

Dr. L. M. Houghton, 74 Church Road, Redfield, Bristol 5.

Dr. H. I. Howard, Health Centre, Leinster Avenue, Bristol 4.

Dr. H. B. Howell, Litfield House, Clifton, Bristol 8.

Dr. T. G. F. Hudson, 97 Pembroke Road, Clifton, Bristol 8.

Dr. F. W. T. Hughes, Chew Magna, Somerset.

Dr. W. Hughes, Stapleton Hospital, Bristol.
Dr. L. C. Hylton, Eglinton, Princes Road, Clevedon.
Dr. W. H. Hylton, Eglinton, Princes Road, Clevedon.
Dr. I. Iles, 87 Pembroke Road, Clifton, Bristol 8.
Dr. W. M. Jablonski, 14 Oakfield Road, Clifton, Bristol 8.
Mr. W. A. Jackman, Pine Trees, Camp Lane, Clapton-in-Gordano, Somerset.
Dr. C. Jahoda, 5 Windsor Terrace, Clifton, Bristol 8.
Mr. J. A. James, Litfield House, Clifton, Bristol 8.
Dr. J. L. James, 6 Belvedere Road, Redland, Bristol 6.
Mr. P. Jardine, Eye Hospital, Bristol 1.
Dr. F. G. Jenkins, 51 Redcliff Hill, Bristol 1.
Dr. P. K. Jenkins, St. Helena, Marine Parade, Clevedon, Somerset.
Mr. D. M. Jones, Pembroke House, Abbots Leigh Road, Leigh Woods, Bristol.
Dr. P. C. Joscelyne, 40 Eastfield, Westbury-on-Trym, Bristol.
Dr. F. E. S. Keiller, Little Silure, Woodspring Avenue, Worlebury, Weston-super Mare.
Dr. G. C. Kelly, 12 Ridgway Road, Long Ashton, Near Bristol.
Dr. W. Pierce Kelly, 6 Elmsleigh Road, Weston-super-Mare.
Dr. G. D. Kersley, 6 The Circus, Bath.
Dr. A. B. Kettle, Langdale, 19a Ellenborough Park North, Weston-super-Mare.
Dr. S. H. Kingston, 3 Whiteladies Road, Bristol 8.
Dr. A. R. Kerr, 35 Westbrook Road, Bristol 4.
Dr. E. J. Lace, 15 Julian Road, Bristol 9.
Dr. M. E. Lace, 15 Julian Road, Bristol 9.
Dr. C. C. Laird, c/o Mardon Sons & Hall, Temple Gate, Bristol.
Dr. P. Legat, Silver Fish Hotel, Knowle, Near Bridgwater, Somerset.
Dr. A. Leitch, 54 Hill Grove, Henleaze, Bristol.
Dr. L. M. Leitch, 40 Eastfield Road, Westbury-on-Trym, Bristol.
Dr. G. E. P. Lee, 564 Fishponds Road, Bristol.
Dr. M. Lennard, 501 Bishport Avenue, Hartcliffe, Bristol 3.
Professor G. G. Lennon, 8 Druid Road, Stoke Bishop, Bristol 9.
Dr. F. J. W. Lewis, Department of Pathology, Southmead Hospital, Bristol.
Dr. Brian R. Little, The Grange, 15 Arundell Road, Weston-super-Mare.
Dr. E. Little, 65 Whitehall Road, Bristol, 5.
Dr. O. C. Lloyd, Department of Pathology, Canynge Hall, Bristol 8.
Dr. Ian A. Lowe, 39 Oakfield Road, Clifton, Bristol 8.
Dr. H. A. R. Loxdale, 30 Elgin Park, Redland, Bristol 6.
Mr. S. D. Loxton, Litfield House, Clifton, Bristol 8.
Dr. G. Lucas, 20 Queen's Road, Bishopsworth, Bristol.
Dr. J. E. Lucas, c/o 132 York Road, Bristol 3.
Mr. K. Lucas, 60 St. John's Road, Clifton, Bristol 8.
Dr. R. P. Lucas, Northways, Fallodon Way, Bristol 9.
Dr. Eileen M. D. McCarthy, 9 Hurle Road, Clifton, Bristol 8.
Dr. B. E. McConnell, 4 Napier Road, Redland, Bristol 6.
Mr. M. P. McCormack, 18 Richmond Hill, Clifton, Bristol 8.
Dr. A. M. McFarlane, Central Health Clinic, Tower Hill, Bristol.
Mr. H. F. T. MacFetridge, The Victoria Eye Hospital, Hereford.
Dr. D. McGowan, Three Oaks, Worlebury Park Road, Weston-super-Mare.
Mr. W. G. MacGregor, University College Hospital, London, W.C.1.
Dr. A. M. MacLachlan, 167 Redland Road, Bristol 6.
Dr. C. J. MacLaren, 2 Oakfield Road, Clifton, Bristol 8.
Dr. A. I. MacLeod, Rutland Lodge, Linden Road, Clevedon, Somerset.
Dr. G. MacLeod, "Nerrylee", Cambridge Road, Clevedon, Somerset.
Mr. A. C. MacPherson, 9 Abbey Road, Westbury-on-Trym, Bristol.
Dr. J. Macrae, Ham Green Hospital, Pill, Near Bristol.
Dr. R. Maggs, Turridon, The Drive, Hellingly, Sussex.
Dr. C. V. M. Mahood, 29 Highbury Road, Bristol 7.
Dr. K. Maksumczyk, 40 Harcourt Road, Redland, Bristol 6.
Dr. K. C. Mallen, 46 Hill Road, Weston-super-Mare.
Dr. Olwen Major, 32 Henleaze Avenue, Bristol.
Dr. John P. Martin, 1 Vicarage Road, Southville, Bristol 3.
Dr. S. F. Marwood, Prospect House, Winterbourne Down, Winterbourne, Bristol.
Dr. J. Mason, 7 Westbury Road, Bristol 9.
Dr. H. G. Mather, 10 Avon Grove, Bristol 9.
Dr. D. Maunsell, 43 Gloucester Road North, Bristol 7.
Dr. J. H. Middlemiss, 11 Pembroke Road, Clifton, Bristol 8.
Mr. A. Miller, Rodney House, Clifton, Bristol 8.
Dr. K. T. W. Miller, St. Vincent's, West Town, Near Bristol.
Dr. C. A. Mills, 16 West Town Avenue, Bristol 4.
Dr. T. Milling, 1 Vicarage Road, Southville, Bristol 3.

Mr. D. M. Milne, Wheatleys, 10 Rayleigh Road, Westbury-on-Trym, Bristol.

Mr. J. P. Mitchell, 23 Parry's Close, Bristol 9.

Dr. F. G. Mogg, 67 Gloucester Road North, Bristol 7.

Dr. S. L. Mohan, 255 Stapleton Road, Bristol.

Dr. R. H. Moore, The Tussocks, Ash Hayes Road, Nailsea, Somerset.

Dr. C. C. Morgans, 65 Lower Redland Road, Bristol 6.

Dr. G. S. Mundy, The Knoll, Chipping Sodbury, Glos.

Dr. F. D. Murphy, 20 Gay Street, Bath.

Dr. Q. St. L. Myles, 7 Apsley Road, Clifton, Bristol 8.

Dr. J. Naish, Algars Manor, Iron Acton, Glos.

Professor A. V. Neale, "Firwood", Long Ashton, Near Bristol.

Dr. A. S. Nethercott, P.O. Impendhle, Natal, South Africa.

Mrs. F. E. Nicholas, "Woodmount", Linden Road, Clevedon, Somerset.

Mr. J. R. Nicholson-Lailey, Beauchamp House, Hatch Beauchamp, Taunton.

Dr. D. Nixon, 12 Luccombe Hill, Redland, Bristol 6.

Dr. J. I. Noble, 32 Whiteladies Road, Bristol 8.

Dr. R. M. Norman, "Sharland", Church Road, Stoke Bishop, Bristol 9.

Dr. Winifred Nott, Fassiefern, Rylestone Grove, Bristol 9.

Dr. D. E. Olliff, Chipping Campden, Glos.

Dr. G. L. Ormerod, 2 Henleaze Road, Bristol 9.

Dr. H. J. Orr-Ewing, 12 Downleaze, Bristol 9.

Dr. B. F. Orton, The Lodge, Warmley, Near Bristol.

Dr. R. W. Orton, The Lodge, Warmley, Near Bristol.

Dr. R. H. Owen, The Leys, Portwall Road, Chepstow, Mon.

Dr. K. Page, 2 Long Cross, Lawrence Weston, Bristol.

Mr. A. G. Palin, Litfield House, Clifton, Bristol 8.

Dr. J. L. Pardoe, Church Cottage, Portishead, Somerset.

Dr. M. Pardoe, Church Cottage, Portishead, Somerset.

Dr. P. W. J. Parkes, 1 Cotham Park North, Bristol 6.

Dr. T. Parr, Harford Lodge, Coombe Dingle, Bristol.

Dr. R. H. Parry, Cefniwrch, Criccieth, Caernarvonshire, N. Wales.

Mr. R. G. Paul, 19 Alexandra Road, Clifton, Bristol 8.

Dr. C. H. Pauli, Batchley Hill, Chew Stoke, Near Bristol.

Dr. M. A. Pauli, Batchley Hill, Chew Stoke, Near Bristol.

Mr. J. H. Peacock, Ellerburn, Hurn Lane, Keynsham, Near Bristol.

Dr. J. E. G. Pearson, Rushmore Grange, West Town, Near Bristol.

Dr. J. F. Pelmore, Frenchay Hospital, Bristol.

Dr. R. Perks, 15 Mortimer Road, Clifton, Bristol 8.

Professor C. B. Perry, Royal Infirmary, Bristol 2.

Mr. D. G. Phillips, Fir Na N'Og, Over Lane, Almondsbury, Glos.

Dr. P. Phillips, 21 Shirehampton Road, Bristol 9.

Dr. G. A. Pilkington, Glebe House, Long Ashton, Somerset.

Dr. A. Pimm, 150 Falcondale Road, Westbury-on-Trym, Bristol.

Dr. J. Pinching, Hillbury, Wells, Somerset.

Dr. A. L. Pirrie, 71 Wellsway, Keynsham, Somerset.

Dr. D. V. Pleasant, 64 Great Brockeridge, Westbury-on-Trym, Bristol.

Mr. J. A. Pocock, 9 Downfield Road, Clifton, Bristol 8.

Dr. B. R. Pollard, Frenchay Hospital, Bristol.

Dr. M. F. Potter, 19 Royal Park, Clifton, Bristol 8.

Dr. H. F. Powell, Ellenborough Park, Weston-super-Mare.

Dr. W. W. Pratt, 1 Hanham Road, Kingswood, Bristol.

Dr. C. H. G. Price, 6 Lansdown Place, Clifton, Bristol 8.

Mr. A. K. Price, Charlcombe Cottage, Lansdown, Bath.

Mr. T. Price, Dawnside, Sion Hill, Bath.

Mr. K. H. Pridie, 58 St. John's Road, Clifton, Bristol 8.

Mrs. K. H. Pridie, 58 St. John's Road, Clifton, Bristol 8.

Dr. R. B. Prowse, "Stevelands", Gloucester Road, Thornbury, Glos.

Dr. D. C. Prowse, Wigmore House, Thornbury, Glos.

Dr. D. Pugh, Dunedin Lodge, Weston Road, Bath.

Dr. H. D. Pyke, 88 Redland Road, Bristol 6.

Dr. A. P. Radford, "Halkyn", Wyck Beck Road, Brentry, Bristol.

Dr. M. Reiss, 1 Windsor Terrace, Clifton, Bristol 8.

Dr. Hilda A. Renolds, 32 Caledonia Place, Clifton, Bristol 8.

Dr. F. B. Richards, 35 Great Brockeridge, Westbury-on-Trym, Bristol.

Dr. J. Ridgway, 18 Blenheim Road, Durdham Down, Bristol.

Dr. P. G. Roads, Central Health Clinic, Tower Hill, Bristol 2.

Dr. A. T. M. Roberts, 12 Southfield Road, Westbury-on-Trym, Bristol.

Dr. J. A. F. Roberts, Clinical Genetics Research Unit, Institute of Child Health, Hospital for Sick Children, Great Ormond Street, London, W.C.1.

Dr. J. A. L. Roberts, 75 High Street, Kingswood, Bristol.
Dr. L. G. Robertson, 162 Redland Road, Bristol 6.
Dr. R. M. Robinson, Weston General Hospital, Weston-super-Mare.
Dr. R. M. Rocke, 4 Goldney Avenue, Clifton, Bristol 8.
Dr. P. J. Roffey, 19a Whatley Road, Clifton, Bristol 8.
Dr. E. Rogan, Central Health Clinic, Tower Hill, Bristol 2.
Dr. C. T. Ross, The Woodlands, Henbury, Bristol.
Dr. F. G. M. Ross, 2 Briercliffe Road, Coombe Dingle, Bristol.
Dr. A. T. Rowley, Connemara, Locking, Weston-super-Mare.
Dr. G. de M. Rudolf, Mount Pleasant, Victoria Road, Clevedon, Somerset.
Dr. L. Sahlmann, Thornbury Park, Thornbury, Glos.
Mr. C. P. Sames, 18 The Circus, Bath.
Dr. O. E. L. Sampson, Woodlands, Parklands Road, Bower Ashton, Bristol 3.
Dr. E Saphier, 12 St. Oswald's Road, Redland, Bristol 6.
Dr. D. H. Sarafian, Moda, Chipping Sodbury, Glos.
Mr. G. Scarff, The Garden House, Coates, Near Pullborough, Sussex.
Mr. T. L. Schofield, Sunnycroft, Bloomfield Park, Bath.
Mr. P. W. Seargeant, 123 Soundwell Road, Staple Hill, Bristol.
Mr. H. L. Shepherd, Litfield House, Clifton, Bristol 8.
Dr. J. W. Shield, White Barns, Bleadon Hill, Weston-super-Mare.
Dr. T. L. H. Shore, Linden Lodge, Haines Hill, Taunton.
Dr. S. Silvey, "Denewood", Coombe Dingle, Bristol.
Dr. D. Simpson, 92 Queen's Road, Clifton, Bristol 8.
Dr. Kenneth Simpson, The Cottage, Pembroke Grove, Clifton, Bristol 8.
Dr. R. E. Simpson, 88 Hampton Road, Bristol 6.
Dr. K. S. Sinclair, 8a Stafford Place, Weston-super-Mare.
Dr. P. S. Sinclair, 520 Filton Avenue, Bristol 7.
Dr. J. Sluglett, 2 Knowle Road, Bristol 4.
Dr. A. L. Smallwood, The Briars, Riding Barn Hill, Wick, Bristol.
Dr. J. Smith, Wentworth House, Wells Road, Bristol 4.
Dr. K. C. P. Smith, 14 Howard Road, Redland, Bristol 6.
Mr H. J. Drew-Smythe, Tockington, Nr. Bristol.
Mr. J. W. E. Snawdon, Litfield House, Clifton, Bristol 8.
Dr. H. K. V. Soltau, 19 The Avenue, Clifton, Bristol 8.
Dr. J. V. Sparks, 11 Claremont Road, Bath.
Dr. J. Spencer-Smith, 290 Passage Road, Brentry, Bristol.
Dr. T. R. Steen, 21 Elgin Park, Redland, Bristol 6.
Dr. R. J. Stephen, 92 Redland Road, Bristol 6.
Dr. G. D. Steven, 6 Upper Church Street, Bath.
Dr. J. M. Stobo, 173 Wells Road, Bristol 4.
Dr. S. A. K. Stokes, Troy House, South Road, Kingswood, Bristol.
Dr. Margaret E. R. Stoneman, Cranford, Primley Road, Sidmouth, Devon.
Dr. A. J. Struthers, 100 Church Road, Redfield, Bristol 5.
Dr. D. Struthers, 165-7 High Street, Street, Somerset.
Dr. G. Struthers, 100 Church Road, Redfield, Bristol 5.
Dr. Joan Struthers, Clouds Hill House, St. George, Bristol.
Dr. H. Summers, 79 Burley Crescent, Downend, Bristol.
Dr. C. L. Sutherland, 37 Shipley Road, Westbury-on-Trym, Bristol.
Dr. F. Sutton, Litfield House, Clifton, Bristol 8.
Major R. J. K. Tallack, Medical Officer, c/o Postmaster, Zanzibar.
Dr. E. A. Tarleton, 493 Long Cross, Lawrence Weston, Bristol.
Mr. D. G. C. Tasker, Downs Edge, Stoke Bishop, Bristol 9.
Dr. A. L. Taylor, 3 Apsley Road, Clifton, Bristol 8.
Mr. A. L. Taylor, Redhurst, 68 Ravelston Dykes, Edinburgh.
Dr. J. T. C. Taylor, Summerleaze, Shirehampton Road, Avonmouth, Bristol.
Mr. J. L. Temple, 23 Cecil Road, Weston-super-Mare.
Dr. H. Temple-Phillips, Central Health Clinic, Tower Hill, Bristol 2.
Dr. S. W. W. Terry, 66 Queen's Court, Clifton, Bristol 8.
Dr. R. C. P. Thomas, 4 Woodbridge Road, Knowle, Bristol 4.
Dr. J. L. G. Thomson, Frenchay Hospital, Bristol.
Dr. G. H. Tovey, Blaise Cottage, Henbury, Bristol.
Dr. L. A. D. Tovey, 47 Druid Hill, Stoke Bishop, Bristol 9.
Dr. K. M. Townend, Heywood, Pill, Near Bristol.
Mr. V. Townrow, 69 Coombe Lane, Bristol 9.
Dr. V. S. Tryon, 3 Harley Place, Clifton, Bristol 8.
Dr. R. C. Tudway, General Hospital, Bristol 1.
Dr. M. D. Turner, 11 Clevedon Road, Weston-super-Mare.
Dr. E. C. Turton, Barrow Hospital, Barrow Gurney, Near Bristol.
Dr. R. C. Tyledesley, 267 Soundwell Road, Kingswood, Bristol.

Dr. N. S. B. Vinter, 267 Soundwell Road, Kingswood, Bristol.

Dr. Patricia Vowles, 19 Southmead Road, Westbury-on-Trym, Bristol.

Dr. M. L. Waite, 56 Queen's Court, Clifton, Bristol, 8.

Professor Milnes Walker, Rockdunder, Wrington, Near Bristol.

Miss S. C. B. Walker, 44 Kewstoke Road, Bristol 9.

Dr. W. L. Walker, Hortham House, Almondsbury, Near Bristol.

Dr. T. B. Wansbrough, 23 Pembroke Road, Clifton, Bristol 8.

Dr. C. E. M. Ware, Taunton & Somerset Hospital, Musgrove Park Branch, Taunton.

Dr. R. P. Warin, 9 Clifton Park, Bristol 8.

Dr. P. K. G. Warren, Stapleton Hospital, Bristol.

Mr. R. Warren, 56 Stanhope Road, Weston-super-Mare.

Mr. P. C. Watson, 3 The Dell, Westbury-on-Trym, Bristol.

Dr G. C. Watt, Priors Mead, Easton-in-Gordano, Somerset.

Dr. A. Weeks, The Firs, Downend, Bristol.

Dr. R. West, Wraxhall House, Wraxall, Somerset.

Dr. P. Westhead, Meadow Cottage, Lye Hole, Redhill, Somerset.

Dr. R. R. Wethered, Englewood, Falcondale Road, Westbury-on-Trym, Bristol.

Dr. R. G. Wilbond, 76 Chesterfield Road, St. Andrew's Park, Bristol 6.

Dr. A. Wilkinson, 3 Ivywell Road, Sneyd Park, Bristol 9.

Dr. I. Williams, "Ardrem", Hill View, Henleaze, Bristol.

Dr. J. I. Williams, 3 Deanery Road, Kingswood, Bristol.

Dr. T. D. Williams, Department of Physiology, The University, Bristol 8.

Dr. J. Willoughby, Wellclose, Nailsea, Somerset.

Mr. M. G. Wilson, Green Gables, Passage Road, Brentry, Bristol.

Dr. T. N. P. Wilton, 24 Great Brockeridge, Westbury-on-Trym, Bristol.

Dr. H. Wohlfeld, 37 The Circus, Bath.

Dr. R. C. Wofinden, 2 Church Road, Stoke Bishop, Bristol 9.

Dr. G. E. Woods, 21 Downs Cote View, Westbury-on-Trym, Bristol.

Dr. W. Woolley, 239 Cranbrook Road, Redland, Bristol 6.

Dr. D. W. Wright, Southmead Hospital, Bristol.

Professor J. M. Yoffey, 1 Tyndall Avenue, Bristol 2.

### SUBSCRIBERS TO THE "JOURNAL"

(NON-MEMBERS)

Dr. H. S. ALLEN, 5 Berkeley Place, Cheltenham.
Dr. H. E. C. BENTLEY, 675 Manchester Road, Bradford, Yorks.
Dr. R. F. BOLT, 4 Gordon Mansions, Torrington Place, London W.C.1.
Dr. J. L. BRIMBLECOMBE, Pranketts, Stoke-under-Ham, Somerset.
Bristol General Hospital, S.R.O.
Dr. J. E. BROWN, Belleville, la Bergerac Road, Maraval, Trinidad, B.W.I.
Dr. W. JOYCE BROWN, Bridge House, Kelvedon, Essex.
Dr. B. CAMERON, 186 Heaton Park Road, Newcastle-upon-Tyne.
Dr. J. R. CHARLES, The Cottage, Churchill, Near Bristol.
Dr. N. C. COOMBS, 55 Galgate, Barnard Castle, Co. Durham.
Dr. C. CORFIELD, 5 Kensington Place, Bristol 8.
Cornwall Clinical Society, Dr. W. L. STEWART, Grampound, Cornwall.
Dr. J. N. P. DAVIES, Department of Pathology, Makerere College, Kampala, Uganda.
Mr. W. J. GALL, No. 2 Lodge, Leicester General Hospital, Leicester.
Dr. H. A. HAMILTON, 9 College Green, Gloucester.
Royal Cornwall Infirmary, Truro, Cornwall.
Dr. R. F. JARRETT, 9 College Green, Gloucester.
Karolinska Institutets Bibliotek, Stockholm 60, Sweden.
Dr. E. F. KING, 79 Harley Street, London, W.1.
Dr. R. A. LATTEY, Amroth, Chelston, Torquay.
Dr. A. LEECH-WILKINSON, 25 The Circus, Bath.
Dr. J. LOWE, St. Margaret's Hospital, Near Swindon.
Dr. P. A. MACCALLUM, Craigmore, Torquay.
McGill University, Medical Library, 3640 University Street, Montreal, Canada.
Dr. I. F. MACMATH, Berryfield Maternity Hospital, Bradford-on-Avon.
Dr. N. E. MELLING, Usk Villa, Sennybridge, Brecon, South Wales.
Professor A. A. MONCRIEFF, Hospital for Sick Children, Great Ormond Street, London W.C.1.
Dr. G. P. O'DONNELL, 17 Green Lane, Redruth, Cornwall.
Sir J. H. PARSONS, 54 Queen Anne Street, London, W.
Dr. D. E. MARGARET PIERCE, Holdsworth Memorial Hospital, Mysore City 1, S. India.
Dr. A. ROBINSON-THOMAS, 20 Devon Square, Newton Abbot.
Dr. A. M. ROSS, 17 Grosvenor Road, Bournemouth.
Royal Cornwall Infirmary, Truro, Cornwall.
Mr. A. H. SANDEMAN, 5 Berkeley Place, Cheltenham.
Dr. J. M. SANSON, 20 Gay Street, Bath.
Dr. C. SEWARD, 20 West Southernhay, Exeter.
Dr. D. K. SOLTAU, 19 The Avenue, Clifton, Bristol 8.
South Devon & East Cornwall Hospital, Greenbank Road, Plymouth, (Hospital Secretary).
Dr. W. N. TRIBE, c/o S.P.G., 15 Tufton Street, London, S.W.1.
Dr. D. M. H. TRIPP, Winthill House, Banwell, Near Weston-super-Mare.
Dr. C. N. VAISEY, Freshford, Near Bath.
Dr. S. C. WAKE, "Stanhope", Bideford, N. Devon.
Dr. C. W. WEDDELL, Bodyville, 46 Miller Street, West Melbourne, Australia.

### Appointments

### UNITED BRISTOL HOSPITALS

*July to September*, 1957

| *Name* | *Appointment* | *Formerly* |
|---|---|---|
| JOLLY, G. F., M.R.C.O.G. | Senior Registrar in Obstetrics and Gynaecology. | Resident Registrar, Southmead Hospital. |
| WALTERS, G., M.B., CH.B., M.R.C.P. | Senior Registrar in Pathology. | Demonstrator in Pathology, University of Bristol. |
| DONNISON, MISS A. B., M.B., B.CH. | Registrar in Paediatrics. | R.M.O. (Registrar Grade), Tadworth Court. |
| COOKSEY, M. W., B.D.S. | Registrar in Dental Surgery. | Resident Dental House Surgeon at St. Bartholomew's Hospital, London. |
| DRINNAN, A. J., B.D.S. | Registrar in Dental Surgery. | S.H.O., Dental Hospital, Bristol. |
| ALEXANDER, D. M., MB., CH.B. | Registrar in Radiodiagnosis. | House Surgeon to General Surgical Unit, Bridge of Earn Hospital, Perthshire. |
| CABLE, R., M.B., CH.B. | Registrar in Radiodiagnosis. | Part-time Registrar, B.R.I. |
| FARRANT, P. C., M.B., CH.B. | Senior Medical Registrar (Research). | S.H.O. in Medicine, Department of Medicine, B.R.I. |

Mr. I. W. Cameron, B.D.S., Dental Registrar, has been appointed Lecturer in Dental Surgery in the University of Bristol.

### SOUTH WESTERN REGIONAL HOSPITAL BOARD

### BRISTOL

| *Name* | *Appointment* | *Formerly* |
|---|---|---|
| BLOTT, KATHLEEN, M.B., CH.B. (BRISTOL), D.A., F.F.A.R.C.S. | Consultant Anaesthetist, Southmead Hospital. | Anaesthetist to the Neurosurgical Unit, Royal Perth Hospital, Perth, Australia. |
| DILWORTH, MISS N. M., M.B., B.S. (ADELAIDE). | Anaesthetic Registrar, Southmead Hospital. | Locum Anaesthetic Registrar, Frenchay Hospital. |
| THOMAS, N. S., M.B., B.S. (LONDON). | Anaesthetic Registrar, Frenchay Hospital. | S.H.O. Anaesthetist, Frenchay Hospital. |
| TODD, C., L.R.C.P., M.R.C.S., D.A. | G.P. Anaesthetist, Weston-super-Mare General Hospital. | Locum Tenens Anaesthetist, Weston-super-Mare Hospital. |
| BIRD, M. W., M.R.C.S., L.R.C.P., D.O.M.S. | Clinical Assistant in Ophthalmology, Wells and District Hospital. | Is a registered Ophthalmic Medical Practitioner under Supplementary Scheme. |
| EASTHAM, R. D., B.A., M.D. (CAMBRIDGE), D.C.P. (LONDON), Diploma of Pathology (London). | Consultant Pathologist, Frenchay Hospital. | Consultant Pathologist, Newcastle General Hospital, in charge of Medical Biochemistry. |
| FOSTER, A. R., B.SC. (LEEDS), M.B., CH.B. (LEEDS). | Registrar, Bristol Mental Hospitals. | S.H.O., Bristol Mental Hospitals. |

### NORTH GLOUCESTERSHIRE

| | | |
|---|---|---|
| OWEN, K. LLOYD, M.R.C.S., L.R.C.P., F.F.A.R.C.S. | Consultant Anaesthetist, North Gloucestershire Clinical Area. | Senior Anaesthetic Registrar, Cardiff Royal Infirmary. |

### *BATH*

| | | |
|---|---|---|
| MURPHY, P., M.B., B.CH., N.U.I., D.A. | Anaesthetic Registrar, Bath Group of Hospitals. | Locum Anaesthetic Registrar, Bath Group of Hospitals. |
| BROTMACHER, L., M.R.C.P., (LONDON), M.D. (LONDON), PH.D. (LONDON). | Senior Medical Registrar, Royal United Hospital, Bath. | Research Fellow, Guy's Hospital. |
| KILPATRICK, MISS L., M.B., CH.B. (EDIN.). | Registrar in Obstetrics and Gynaecology, Bath Group of Hospitals. | S.H.O. in Surgery and Gynaecology, Queen Mary's Hospital, Sidcup. |

### *DEVON AND CORNWALL*

| | | |
|---|---|---|
| KENDALL, J. G., M.B., CH.B. (B'HAM), F.R.C.S. (ENGLAND). | Consultant Orthopaedic Surgeon, West Cornwall Clinical Area. | Senior Registrar, The Prince of Wales Orthopaedic Hospital, Cardiff. |
| PARKER, JOHN, M.A., M.B., B.CHIR (CANTAB), M.R.C.P. (LONDON). | Re-appointed for further year as Clinical Assistant in Paediatrics in Torquay (Torbay and Rosehill Children's Hospitals). | Is in general practice, Torquay |
| COUPER, L., M.B., CH.B. (GLASGOW), D.P.M. (MANCHESTER). | Medical Superintendent, Digby-Wonford Hospital, Exeter. | Consultant Psychiatrist and Deputy Superintendent, Winwick Hospital, Nr. Warrington. |
| PRICE, J. H., M.B., B.S. (LONDON), D.P.M. (LONDON) PT. I. | Senior Psychiatric Registrar, Moorhaven Hospital, Ivybridge. | Psychiatric Registrar, Hill End Hospital, St. Albans. |

### *1957 Index to Vol. 72*

Alexander, G. L.—Head Injuries, 91

Astrocytoma treated by Radiotherapy: Death from Acute Leukaemia.—a Clinical Pathological Conference, 67

Better Health at Work, Towards.—T. G. Faulkner Hudson, 117

Blue to Pink, From.—E. G. Bradbeer, 46

Bradbeer, E. G.—From Blue to Pink, 46

Bristol and Bordeaux.—J. A. Cosh, 98

British West Indies, Some Notes of a Medical Visitor to the—Prof. A. V. Neale, 33

Child Art.—D. E. Milner, 125

Clinical Pathology and General Practice.—H. J. Gibson, 109

Clinical Pathological Conferences:
—Diabetes Mellitus with Renal Papillary Necrosis, 26
—Astrocytoma treated by Radiotherapy, 67
—Sarcoidosis treated with Cortisone, 101
—Acute Necrosis of the Liver in Pregnancy, 129

Cosh, J. A.—Bristol and Bordeaux, 98

Diabetes Mellitus with Renal Papillary Necrosis, 26

Doctor's Visit to U.S.S.R.—A. M. MacLachlan, 1

Eyre-Brook, A. L.—A Tour of the South-Western Pacific, 75

Faulkner Hudson, T. G.—Towards Better Health at Work, 117

Flavell Matts, S. G.—Symptoms and Treatment of Acute Follicular Tonsilitis, 63

Gibson, H. J.—Clinical Pathology and General Practice, 109

Head Injuries.—G. L. Alexander, 91

Hemphill, R. E.—The Use and Misuse of Sedatives and Hypnotic Drugs, 12

MacLachlan, A. M.—A Doctor's Visit to U.S.S.R., 1

Milner, D. E.—Child Art, 125

Neale, Prof. A. V.—Some Notes of a Medical Visitor to the British West Indies, 33

Necrosis, Acute, of the Liver in Pregnancy—a Clinical Pathological Conference, 129

Perry, Prof. C. Bruce.—The Prevention of Acute Rheumatism, 86

Port Health Service, The.—D. T. Richards, 56

Public Analyst, The, and the New Food and Drugs Act 1955.—E. G. Whittle, 23

Rheumatism, The Prevention of Acute.—Prof. C. Bruce Perry, 86

Richards, D. T.—The Port Health Service, 56

Saroidosis Treated with Cortisone—a Clinical Pathological Conference, 101

Sedatives and Hypnotic Drugs, The Use and Misuse.—R. E. Hemphill, 12

South-Western Pacific, A. Tour of the—A. L. Eyre-Brook, 75

Symptoms and Treatment of Acute Follicular Tonsilitis, The.—S. G. Flavell Matts, 63

Whittle, E. G.—The Public Analyst and the New Food and Drugs Act 1955, 23

